# Association Study Among Candidate Genetic Polymorphisms and Chemotherapy-Related Severe Toxicity in Testicular Cancer Patients

**DOI:** 10.3389/fphar.2019.00206

**Published:** 2019-03-08

**Authors:** María A. Lavanderos, Juan P. Cayún, Ángela Roco, Christopher Sandoval, Leslie Cerpa, Juan C. Rubilar, Roberto Cerro, Sebastián Molina-Mellico, Cesar Celedón, Berta Cerda, Elena García-Martín, José A. G. Agúndez, Cristián Acevedo, Karina Peña, Dante D. Cáceres, Nelson M. Varela, Luis A. Quiñones

**Affiliations:** ^1^Laboratory of Chemical Carcinogenesis and Pharmacogenetics, Department of Basic and Clinical Oncology, Faculty of Medicine, University of Chile, Santiago, Chile; ^2^Servicio Metropolitano de Salud Occidente, Santiago, Chile; ^3^Instituto Nacional del Cáncer, Santiago, Chile; ^4^Institute of Molecular Pathology Biomarkers, ARADyAL, University of Extremadura, Cáceres, Spain; ^5^Clinical Hospital University of Chile, Santiago, Chile; ^6^Department of Oncology, Hospital San Juan de Dios, Santiago, Chile; ^7^Instituto de Salud Poblacional, Facultad de Medicina, Universidad de Chile, Santiago, Chile

**Keywords:** pharmacogenetics, polymorphisms, toxicity, testicular cancer, ADRs

## Abstract

Testicular cancer is one of the most commonly occurring malignant tumors in young men with fourfold higher rate of incidence and threefold higher mortality rates in Chile than the average global rates. Surgery is the initial line of treatment for testicular cancers, and is generally followed by chemotherapy, usually with combinations of bleomycin, etoposide, and cisplatin (BEP). However, the adverse effects of chemotherapy vary significantly among individuals; therefore, the present study explored the association of functionally significant allelic variations in genes related to the pharmacokinetics/pharmacodynamics of BEP and DNA repair enzymes with chemotherapy-induced toxicity in BEP-treated testicular cancer patients. We prospectively recruited 119 patients diagnosed with testicular cancer from 2010 to 2017. Genetic polymorphisms were analyzed using PCR and/or qPCR with *TaqMan*^®^probes. Toxicity was evaluated based on the Common Terminology Criteria for Adverse Events, v4.03. After univariate analyses to define more relevant genetic variants (*p* < 0.2) and clinical conditions in relation to severe (III–IV) adverse drug reactions (ADRs), stepwise forward multivariate logistic regression analyses were performed. As expected, the main severe ADRs associated with the non-genetic variables were hematological (neutropenia and leukopenia). Univariate statistical analyses revealed that patients with *ERCC2* rs13181 T/G and/or *CYP3A4* rs2740574 A/G genotypes are more likely to develop alopecia; patients with *ERCC2* rs238406 C/C genotype may develop leukopenia, and patients with *GSTT1*-null genotype could develop lymphocytopenia (III–IV). Patients with *ERCC2* rs1799793 A/A were at risk of developing severe anemia. The *BLMH* rs1050565 G/G genotype was found to be associated with pain, and the *GSTP1* G/G genotype was linked infection (*p* < 0.05). Multivariate analysis showed an association between specific *ERCC1/2* genotypes and cumulative dose of BEP drugs with the appearance of severe leukopenia and/or febrile neutropenia. Grades III–IV vomiting, nausea, and alopecia could be partly explained by the presence of specific *ERCC1*/2, *MDR1*, *GSTP1*, and *BLMH* genotypes (*p* < 0.05). Hence, we provide evidence for the usefulness of pharmacogenetics as a tool for predicting severe ADRs in testicular cancer patients treated with BEP chemotherapy.

## Introduction

Testicular cancers are malignant cancers that mainly affect young men. Cisplatin-based chemotherapy has been routinely used as the standard therapy for the treatment of metastatic testicular cancers. The standard treatment scheme for patients with low-risk testicular cancers involves three cycles of bleomycin, etoposide, and cisplatin (BEP) or four cycles of etoposide and cisplatin. Patients with intermediate-risk or high-risk disease are routinely treated with up to four cycles of BEP or four cycles of etoposide, ifosfamide, and cisplatin. Chemotherapy for rescuing the relapse of testicular cancers includes a standard dose of vinblastine, cisplatin and ifosfamide (Pizzocaro et al., 1985; Mezvrishvili and Managadze, 2006).

However, chemotherapy represents a significant challenge in the day-to-day management of the patients, since the inter-individual variations in response to the chemotherapy drugs are a major cause of concern. A drug that is well-tolerated and causes a strong response in some patients could prove to be ineffective, toxic or cause adverse drug reactions (ADRs) in others; therefore, research is required to analyze the effects of genetic variations on the pharmacokinetics and/or pharmacodynamics of these drugs. Statistics indicate that 1 in 15 hospital admissions for testicular cancer in the United Kingdom was due to ADRs (Pirmohamed et al., 2004), and adverse drug effects in hospitalized patients were identified to be the fifth leading cause of death in the United States (Mancinelli et al., 2000). Other evidence suggests that the annual number of reported cases of adverse reactions is around two millions which has been reported to cost US $100 billion (Ross et al., 2011). The antineoplastic drugs have often been shown to exhibit toxicity at therapeutic concentrations; therefore, ADRs are frequently observed during chemotherapy, which has reportedly increased the total medical costs by 1.9%, and the costs of medicines by 15% (Huang and Ratain, 2009).

Response to chemotherapy may be determined by gene polymorphisms, which eventually govern the metabolism of cytotoxic drugs. The allelic variants of genes related with pharmacokinetics/pharmacodynamics processes can alter the enzyme activity of the metabolic proteins leading to changes in drug metabolism (Agundez, 2004; Quiñones et al., 2017). Therefore, the response to chemotherapy in patients may be partly determined by gene polymorphisms involved in the metabolism of these cytotoxic drugs. Most of the chemotherapeutic drugs are metabolized by phase I polymorphic Cytochrome P450 enzymes, whose variant alleles commonly affect drug effectiveness and toxicity (Kivistö et al., 1995; Quiñones et al., 2008; Božina et al., 2009; Ingelman-Sundberg and Sim, 2010; Deenen et al., 2011). Cytochrome P450 isoforms 3A4 and 3A5 (CYP3A4/5) play a role in etoposide metabolism (Zhuo et al., 2004). The *CYP3A4^∗^1B* rs2740574 genotype is associated with an increased risk of leukemia following treatment with etoposide and teniposide. This variant has been reported to contribute to higher risk of secondary cancers (Felix et al., 1998). Moreover, two studies have also suggested that *CYP3A4^∗^1B* is a risk factor allele for prostate cancer (Keshava et al., 2004).

On the other hand, polymorphisms in phase II enzymes have been reported to affect the resistance and adverse reactions to several chemotherapy regimens (Jain et al., 2007; Mazerska et al., 2016; Marchewka et al., 2017). Previous reports have shown that Glutathione-*S*-transferases (GSTs) are associated with resistance to cisplatin-based chemotherapy (Roco et al., 2014; Nissar et al., 2017). *GSTM1*-null and/or *GSTT1*-null genotypes are associated with the development of grades III–IV thrombocytopenia (Cho et al., 2010) upon combined chemotherapy with rituximab and cyclophosphamide/doxorubicin/vincristine/prednisone or R-CHOP. Grade ≥ III toxicity and grade ≥ III neurotoxicity has been observed in children with medulloblastoma treated with cycles of cisplatin, cyclophosphamide, and vincristine (Barahmani et al., 2009). Besides, the *GSTP1* Ile105Val polymorphism has been strongly associated with progression-free survival. The T/T genotype of the −69 C > T *GSTA1* polymorphism correlates with overall survival. Thrombocytopenia, anemia, and neuropathy were less frequent among patients with the *GSTM1*-null or *GSTM3* intron 6 AGG/AGG genotypes (Khrunin et al., 2010). Moreover, the presence of UDP-Glucuronosyl-transferase Family 1 Member A1 (*UGT1A1*) polymorphic variants has also been associated with chemotherapy response and Gilbert Syndrome after chemotherapy (Ha et al., 2017; Negoro et al., 2018). The most studied *UGT1A1* allele is *UGT1A1^∗^28*, which has mainly been associated with an increased risk of irinotecan toxicity (Lyer et al., 2002). In line with this evidence, the FDA recommended tests to detect the presence of *UGT1A1^∗^28*, to predict patients at risk of irinotecan poisoning^1^.

ABC-drug transporters are also implicated in the metabolic response to chemotherapy (Domenichini et al., 2019). P-glycoprotein 1 (P-gp1), also known as multidrug resistance protein 1 (MDR1) or ATP- binding cassette sub-family B member 1, is highly polymorphic and several studies have reported that carriers of the T-allele for the genetic variation *C3435T* (rs1045642) have an increased risk of colon, breast, and renal cancer (Phuthong et al., 2017). However, Cizmarikova et al. (2010), found no significant differences in hematological toxicities in the groups with the *MDR1 C3435T* polymorphism in breast cancer.

On the other hand, bleomycin (BLM) is metabolically inactivated by the action of cysteine peptidase bleomycin hydrolase (BLMH) (Schwartz et al., 1999; Deenen et al., 2011). de Haas et al. (2008), showed that *BLMH* A/G genotype was related with reduced survival and higher prevalence of early relapses in testicular cancer patients. Recently, Jóna et al. (2016), showed lower rate of pulmonary complications in the A/A genotype group than those in the group containing the mutated allele: A/G+G/G in ABVD (doxorubicin, bleomycin, vinblastine, and dacarbazine)-treated Hodgkin lymphoma patients.

Several antineoplastic drugs have been reported to cause DNA damage. Numerous studies have investigated the association between single nucleotide polymorphisms (SNPs) in DNA repair enzymes, clinical outcomes, and resistance to chemotherapy (Zamble and Lippard, 1995; Gossage and Madhusudan, 2007; Frosina, 2009; Zhang et al., 2017) indicating that reduced activity of DNA repair enzymes may lead to an improved response to chemotherapy. However, compromised repair may also lead to accumulation of DNA damage in normal cells, leading to adverse side effects in normal tissues, thereby predisposing them toward secondary cancers. Due to these side effects, commonly used chemotherapeutic agents, including alkylating agents (cisplatin), inhibitors of DNA topoisomerase II (etoposide), and BLM have proven to be toxic to the patients.

Excision repair cross-complementary 1 (ERCC1) is a key protein involved in nucleotide excision repair (NER), and ERCC1-Xeroderma pigmentosum (ERCC1-XPF) catalyzes the incision on the site of DNA damage (Park et al., 1995). Elevated ERCC1 expression in cancers is associated with resistance to DNA damage-based chemotherapy (Chiu et al., 2011; Tsai et al., 2011). On the other hand, Xeroderma pigmentosum complementary group D (*XPD/ERCC2*) encodes a helicase which participates in both NER and basal transcription as part of the transcription factor IIH. Mutations abolishing the enzymatic function of the ERCC2 protein are manifested clinically in combinations of three severe syndromes, including Xeroderma pigmentosum (Lehmann, 2001; Clarkson and Wood, 2005). Polymorphisms in these enzymes further affect DNA repair and are involved in resistance to chemotherapy, survival, and cancer manifestation (Benhamou and Sarasin, 2002; Giovannetti et al., 2011).

Based on the accumulated scientific evidence about BEP chemotherapy, we here focused on functionally significant gene polymorphisms in proteins that control metabolism, uptake, and response to BEP drugs (^2^Roco et al., 2014; Chen et al., 2017). Mainly, the allelic variants of *CYP3A4* rs2740574 *(^∗^1B)*, *CYP3A4* rs35599367 *(^∗^22)*, *CYP3A5* rs776746 *(^∗^3)*, *GSTM1*-null, *GSTP1* rs1695, *GSTT1*- null, *UGT1A1* rs8175347 *(^∗^28)*, *BLMH* rs1050565*, ERCC1* rs11615*, ERCC1* rs3212986, *ERCC2* rs13181, *ERCC2* rs1799793, *ERCC2* rs238406, and *MDR1* rs1045642 were analyzed with non-genetic factors to validate their association with ADRs in testicular cancer patients treated with BEP schedule.

## Materials and Methods

### Patients

One hundred nineteen (119) patients with germinal (seminoma or non-seminoma) testicular cancer confirmed histologically, >18 years old, without chronic unbalanced or systemic pathology or other active cancers and without being included in the interventional study conducted 3 months before, were enrolled prospectively in this study. The enrollment was carried out from December 2010 – December 2017 at the Polyclinic of Hematology-oncology of Hospital San Juan de Dios, and the Polyclinic of Oncology of the National institute of Cancer. All the patients signed a written consent and an agreement to be included in this study. The study was carried out under strict ethical procedures recommended by the Ethics Committee of the University of Chile (August 17, 2010), and the Northern Metropolitan Health Service, National Cancer Institute (April 4, 2015), in accordance with the procedures suggested in the Declaration of Helsinki (Declaration of Helsinki, 1964), and according to Chilean Laws 20.120, 20.584, and 19.628, and the guidelines of the Good Clinical Practices. Chemotherapy regimen for all patients involved the administration of cisplatin and etoposide in combination with bleomycin for up to four cycles (BEP schedule), which is the standard treatment for patients with low or intermediate risk testicular cancers, all over the world (Pizzocaro et al., 1985; Mezvrishvili and Managadze, 2006).

This regimen was applied in conjunction with a rigorous and standardized hydration regimen for all the patients. The clinical variables were obtained from patients’ clinical files and recorded in proper case report forms (CRFs). Laboratory assessment was performed after each cycle of chemotherapy. Treatment-related toxicity was graded according to the terminological common criteria for adverse reactions (v4.03), of the U.S. Department of Health and Human Services with a follow-up after 6 months of the last cycle of chemotherapy. The association study included age, height, weight, body surface area, and cumulative doses for the statistical analyses. Table 1 shows the general characteristics of the studied patients.

**Table 1:** Baseline characteristics of patients.

	*n* = 119	%
Age, years		
Average ± SD (range)	28.05 ± 8.29 (16–56)	
Median	27	
BSA, m^2^		
Average ± SD (range)	1.89 ± 0.19 (1.5–2.8)	
Median	1.86	
Histologic type		
Seminoma	16	13.45
No seminoma	103	86.55
Number of cycles		
2 cycles	31	26.05
3 cycles	49	41.18
4 cycles	38	31.93
5 cycles	1	0.84
Cisplatin dose per cycle day		
Mg per BSA	100	
Etoposide dose per cycle day		
Mg per BSA	120	
Bleomycin dose per cycle day		
UI	30	

*BSA, body surface area; SD, standard deviation.*

### Genotyping Analysis

Potentially functional SNPs encoding the proteins related to BEP response were obtained from the NCBI dbSNP database^3^, and the SNPinfo Web Server^4^ based on the level of evidence for each SNP (Supplementary Table S1). Genomic DNA was isolated from the peripheral blood samples of the subjects using High Pure PCR Template Preparation Kit (Catalog Number, 11796828001; Roche Diagnostics GmbH, Mannheim, Germany). *CYP3A4^∗^1B* rs2740574, *CYP3A4^∗^22* rs35599367, *CYP3A5^∗^3* rs776746, *UGT1A1^∗^28* rs8175347, *BLMH* rs1050565, *GSTP1* rs1695, *ERCC1* rs11615, *ERCC1* rs321986, *ERCC2* rs13181, *ERCC2* rs238406, *ERCC2* rs1799793, and *MDR1* rs1045642 were analyzed using *TaqMan*^®^SNP Genotyping Assay (Catalog number, 4362691; Thermo Fisher Scientific, Waltham, MA, United States), in an Stratagene Mx3000p real-time PCR system (Agilent Technologies, Santa Clara, CA, United States). The presence of the *GSTM1*-null genotype was determined by the absence of a 273 bp fragment product in a 2% agarose gel (Bio-Rad Laboratories, Hercules, CA, United States). The presence of the *GSTT1*-null genotype was determined by the absence of a 268 bp fragment. Amplification fragment for β-globin was used as the internal control (Quiñones et al., 1999; Roco et al., 2012). Heterozygous and homozygous non-null individuals could not be differentiated, therefore double null genotypes (−/−) are the null genotypes reported. For Quality Assurance purposes we randomly choose 20% of the samples for (a) repetition of the analysis and (b) PCR-RFLP analysis for coincidence. When analyses were not coincident we excluded the samples. The sequences for *TaqMan*^®^probes and primers for PCR are listed in Supplementary Tables S2, S3 enlists the description of each polymorphism.

### Statistical Analyses

We performed a logistic regression analysis using Stata software, version 12.0 (Copyright 1985–2011 StataCorp., LP, College Station, TX, United States). A *p*-value of ≤ 0.05 was considered statistically significant. The odds ratio (OR) and 95% confidence intervals (CI) were reported in the univariate and multivariate logistic regression models. The logistic multivariate models were adjusted stepwise using a forward procedure with *p*-value ≤ 0.2 to include potentially relevant variables in order to derive statistical association models, characterized by Pseudo R^2^. All association studies were assayed by testing three genetic models of inheritance, i.e., dominant, codominant and recessive models, and choosing parameters with better statistical association for each analysis.

For the univariate and multivariate analyses, we define several alternatives for dosage: *Ranges for dose*: to get the ranges we used quartiles (Q) to divide data in four groups, with lower range comprising Q_0_ to the average between Q_1_ and Q_2_, intermediate range comprising of the average between Q_1_ and Q_2_ to the average between Q_2_ and Q_3_ and the largest range comprising of the average between Q_2_ and Q_3_ to Q_4_. *Cumulative dose* was defined as the total dose administered to the patient during all the cycles of chemotherapy. *Cumulative dose by average*: the patients were divided in two groups according to their were lower/equal in relation the average cumulative dose (<average) or higher than the average cumulative dose (>average). Similar procedure was performed for weight, height, body surface and age and for chemotherapy cycles with frequency of 1–2, 3 and 4–5 cycles, getting dummy variables for the statistical analyses.

We did not check Hardy–Weinberg equilibrium (HWE) of our sample because it does not accomplish the conditions for HWE. This is not a random sampling in a random-mating population, a control or general population (Namipashaki et al., 2015) and is a group with a selection bias by the disease (i.e., SNPs can also be related to the cancer).

## Results

### Patient Characteristics

The baseline characteristics of patients are shown in Table 1. A total of 119 Chilean patients from two hospitals in Chile were included and analyzed. Most patients were young (average age: 28.05 years), and were administered 2–5 cycles of BEP and predominantly showed no seminoma (86.55%). The genotypic and allelic frequencies for the analyzed polymorphisms are shown in Supplementary Table S4.

### Toxicity to Chemotherapy

Adverse drug reactions represented in Table 2 were recorded to determine the acute toxicity in patients with testicular cancer treated with BEP chemotherapy. The ADRs are shown in two columns, any grades (I–IV) column and severe or high-grade toxicities (III–IV) column. The most frequent toxicities observed included vomiting (82.35%), nausea (79.83%), anemia (60.68%), neutropenia (53.45%), and alopecia (52.94%). Most frequently observed high-grade toxicities included neutropenia (39.66%), leukopenia (12.71%), febrile neutropenia (12.61%), and vomiting (9.24%).

**Table 2:** Adverse drug reactions (ADRs) according degree of severity^∗^.

	Any grades (I–IV)		Severe (grades III–IV)	
	No.	%	No.	%
**Toxicity**				
Vomiting	98	82.35	11	9.24
Nausea	95	79.83	8	6.72
Anemia	71	60.68	3	2.56
Neutropenia	62	53.45	46	39.66
Alopecia	63	52.94	5	4.20
Leukopenia	49	41.53	15	12.71
Pain	44	36.97	5	4.20
Mucositis	26	21.85	1	0.84
Diarrhea	24	20.17	0	0.00
Dermatological reaction	23	19.33	1	0.84
Thrombocytopenia	21	17.95	2	1.71
Hypotension	19	15.97	0	0.00
Lymphocytopenia	13	11.21	3	2.59
Neurotoxicity	10	8.40	2	1.68
Constipation	8	6.72	0	0.00
Pyrosis	5	4.20	0	0.00
Ototoxicity	4	3.36	0	0.00
Febrile Neutropenia	–	–	15	12.61

*^∗^ADR, adverse drug reaction, evaluated with Common Terminology Criteria for Adverse Events [CTCAE], 2017.*

### Association Between Genotypes and Toxicities

We performed univariate logistic regression of risk for severe (III–IV) ADRs in association with genotypes, in three models of inheritance namely, recessive, codominant, and dominant. The results are shown in Supplementary Table S5 where only results with *p*-value ≤ 0.2 are included for the stepwise forward procedure for multivariate analysis. In Table 3, only statistically significant results for the univariate logistic regression analysis of risk of severe ADRs (III–IV), according to genotypes are shown. These results show that *ERCC2* rs1799793 A/A genotype was associated with anemia in a recessive model of inheritance, *ERCC2* rs13181 T/G and *CYP3A4* rs2740574 A/G genotypes were associated with alopecia in a codominant model of inheritance, and *ERCC2* rs238406 A/A genotype was associated with leukopenia, both in codominant and recessive models of inheritance. *GSTT1*-null genotype was associated with lymphocytopenia, *BLMH* rs1050565 G/G genotype was linked with pain in a recessive model of inheritance and *GSTP1* rs1695 G/G genotype was associated with infections in a recessive model of inheritance.

**Table 3:** Univariate logistic regression analysis of risk of severe ADRs (III–IV) according to genotypes.

ADR^∗^	*n*	OR^∗∗^	95% IC^∗∗∗^	*p*-value^∗∗∗∗^
**Anemia**				
ERCC2 (rs1799793)				
G/G + G/A	110	1.00		Reference
A/A	3	27.00	1.68–434.44	0.020
**Leukopenia**				
ERCC2 (rs238406)				
C/C	58	1.00		Reference
C/A	29	3.82	0.84–17.28	0.082
A/A	26	5.50	1.26–24.10	0.024
ERCC2 (rs238406)				
C/C	58	1.00		Reference
C/A + A/A	55	4.58	1.20–17.45	0.026
**Lymphocytopenia**				
GSTT1				
No null	108	1.00		Reference
Null	4	17.67	1.23–252.73	0.034
**Alopecia**				
CYP3A4^∗^1B (rs2740574)				
A/A	106	1.00		Reference
A/G	12	6.87	1.02–46.06	0.047
G/G	1	–		
ERCC2 (rs13181)				
T/T	77	1.00		Reference
T/G	32	10.86	1.16–101.35	0.036
G/G	6	–		
**Pain**				
BLMH (rs1050565)				
A/A + A/G	93	1.00		Reference
G/G	26	16.73	1.78–157.15	0.014
**Infections**				
GSTP1 (rs1695)				
A/A + A/G	99	1.00		Reference
G/G	18	12.25	1.05–143.09	0.046

*^∗^ADR, adverse drug reaction, evaluated with CTCAE4.03. ^∗∗^OR, odds ratio. ^∗∗∗^95% CI, 95% confidence interval. ^∗∗∗∗^Only statistical significant associations are shown (*p* ≤ 0.05).*

The same analysis was performed for non-genetic factors (e.g., age, sex, weight, height, body surface, cycles, and cumulative dose) (Supplementary Table S6). Table 4 shows only the statistically significant results obtained from the univariate logistic regression analysis of risk of severe ADRs (III–IV), analyzed according to non-genetic factors. We observed that the cumulative dose of bleomycin; bleomycin dose by average or bleomycin cycles by range were associate with febrile neutropenia as well as cisplatin dose by range. Similarly, cumulative total etoposide dose or dose by average were associated with leukopenia as well as the cumulative dose of cisplatin. Besides, neutropenia was associated with both, cumulative or cumulative by average dose of cisplatin or etoposide, and cumulative bleomycin. Finally, alopecia was significantly associated only with cumulative and cumulative by average dose of bleomycin.

**Table 4:** Univariate logistic regression analysis of risk of severe ADRs (III–IV) according to non-genetic factors.

ADR^∗^	*n*	OR^∗∗^	95% IC^∗∗∗^	*p*-value^∗∗∗∗^
**Febrile neutropenia**				
Bleomycin cumulative dose	117	1.01	1.00–1.02	0.014
Bleomycin cumulative dose by average				
≤Average	81	1.00		Reference
>Average	36	3.02	1.00–9.11	0.050
Chemotherapy cycles				
1–2	31	1.00		Reference
3	49	3.41	0.38–30.66	0.274
4–5	39	9.00	1.07–75.51	0.043
**Leukopenia**				
Cisplatin cumulative dose	116	1.00	1.00–1.01	0.017
Etoposide cumulative dose	116	1.00	1.00–1.00	0.006
Etoposide cumulative dose by average				
≤Average	62	1.00		Reference
>Average	54	5.62	1.49–21.15	0.011
**Neutropenia**				
Cisplatin cumulative dose	114	1.00	1.00–1.00	0.037
Etoposide cumulative dose	114	1.00	1.00–1.00	0.041
Cisplatin cumulative dose by average				
≤Average	58	1.00		Reference
>Average	56	2.82	1.29–6.14	0.009
Etoposide cumulative dose by average				
≤Average	60	1.00		Reference
>Average	54	2.72	1.26–5.91	0.011
**Alopecia**				
Bleomycin cumulative dose	117	1.02	1.00–1.03	0.046
Bleomycin cumulative dose by average				
≤Average	81	1.00		Reference
>Average	36	10.00	1.08–92.94	0.043

*^∗^ADR, adverse drug reaction, evaluated with CTCAE4.03. ^∗∗^OR, odds ratio. ^∗∗∗^95% CI, 95% confidence interval. ^∗∗∗∗^Only statistical significant associations are shown (p ≤ 0.05).*

After stepwise forward procedure, using associations with a *p* < 0.2, multivariate logistic regression analyses for the risk of severe ADRs, including genetic and non-genetic factors, were performed. Table 5 show only statistically significant association models for severe ADRs. We obtained significant models for febrile neutropenia, leukopenia, vomiting, nausea, and alopecia.

**Table 5:** Multivariate logistic regression analysis and risk of severe ADRs (grades III–IV), after stepwise forward procedure (cut-off *p* < 0.2).

ADR^∗^	OR^∗∗^	95% IC^∗∗∗^	*p*-value^∗∗∗∗^	Model data
**Febrile neutropenia**				
ERCC1 (rs11615)				Number of obs: 106 *p*-value: 0.0231 Pseudo R^2^: 0.0956
C/C + C/T	1.00		Reference	
T/T	4.89	1.06–22.56	0.042	
Bleomycin cumulative dose	1.01	1.00–1.02	0.028	
**Leukopenia**				
ERCC2 (rs238406)				Number of obs: 111 *p*-value: 0.0031 Pseudo R^2^: 0.1372
C/C	1.00		Reference	
C/A + A/A	4.09	1.04–15.99	0.043	
Etoposide cumulative dose by average				
≤Average	1.00		Reference	
>Average	4.48	1.15–17.48	0.031	
**Vomiting**				
MDR1 (rs1045642)				Number of obs: 111 *p*-value: 0.0121 Pseudo R^2^: 0.1231
CC + CT	1.00		Reference	
TT	4.90	11.14–21.09	0.033	
ERCC1 (rs 3212986)				
C/C	1.00		Reference	
C/A + A/A	0.20	0.04–0.85	0.030	
**Nausea**				
GSTP1 (rs1695)				Number of obs: 115 *p*-value: 0.0344 Pseudo R^2^: 0.1278
A/A + A/G	1.00		Reference	
G/G	5.43	1.04–28.42	0.045	
Cisplatin cumulative dose	1.00	1.00–1.01	0.047	
**Alopecia**				
BLMH (rs 1050565)				Number of obs: 115 *p*-value: 0.0124 Pseudo R^2^: 0.2135
A/A + A/G	1.00		Reference	
G/G	6.95	1.00–48.23	0.050	
ERCC2 (rs13181)				
T/T	1.00		Reference	
T/G + G/G	10.57	1.07–104.02	0.043	

*^∗^ADR, adverse drug reaction, evaluated with CTCAE4.03. ^∗∗^OR, odds ratio. ^∗∗∗^95% CI, 95% confidence interval. ^∗∗∗∗^Only statistical significant association models are shown (*p* ≤ 0.05). Resulting significant equations:*$\mathrm{Febr.Neutropenia}=\frac{p}{1-p}=e^{-\mathrm{5.97}+\mathrm{1.59}[\mathrm{ERCC1 rs11615 C}>T]+\mathrm{0.012[BLM C. DOSE]}}$$\mathrm{Leukopenia}=\frac{p}{1-p}=e^{-\mathrm{3.74}+\mathrm{1.41}[\mathrm{ERCC2 rs238406 C}>A]+\mathrm{1.50[ETOP C. DOSE BY AV]}}$$\mathrm{Vomiting}=\frac{p}{1-p}=e^{-1.\mathrm{88}+\mathrm{1.59}[\mathrm{MDR1}\;\mathrm{rs1045642 C}>T]-\mathrm{1.61}[\mathrm{ERCC1 rs3212986C}>A]}$$\mathrm{Nausea}=\frac{p}{1-p}=e^{\mathrm{5.01}+\mathrm{1.69}[\mathrm{GSTP1 rs1695A}>G]+\mathrm{0.003}[\mathrm{CISP C. DOSE}]}$$\mathrm{Alopecia}=\frac{p}{1-p}=e^{-\mathrm{5.19}+\mathrm{1.94}[\mathrm{BLMH rs1050565 A}>G]+\mathrm{2.36}[\mathrm{ERCC2 rs13181T}>G].}$

## Discussion

Patient response to chemotherapy has been investigated for long, and ADR after chemotherapy is a substantial clinical problem. For testicular cancers, this is particularly relevant since besides surgery (inguinal orchiectomy) chemotherapy is routinely administered with combination of three cytostatic drugs, bleomycin, etoposide, and cisplatin. Even though chemotherapy is quite successful in the treatment for patients with seminoma and the success rates exceed 90%, adverse reactions are frequently observed in response to one of the drugs or the drug combination. Therefore, in the present study, we have evaluated the role of genetic polymorphisms and other non-genetic factors as potential modifying risk factors for ADRs.

In the univariate analyses (Table 3), we found interesting association between *BLMH* rs1050565 G/G genotype and severe pain in patients (OR = 16.73, CI = 1.78–157.15, *p*-value = 0.014). Our observation is in line with the report from White and coworkers who showed an association between acute chest pain and bleomycin infusion (White et al., 1987). Considering that G allele of *BLMH* leads to the incorporation of 443Val in the enzyme, reducing its biochemical activity, this association supports our finding and high bleomycin plasma levels in patients can be expected.

On the other hand, patients with *CYP3A4* rs2740574 A/G genotypes are more likely to develop alopecia (OR = 6.87, CI = 1.02–46.06, *p*-value = 0.047). This gene encodes for the main enzyme involved in etoposide metabolism^2^, and metabolizes cisplatin or bleomycin. The presence of G allele leads to reduced transcription of the enzyme, suggesting a relationship between dose and increased plasma levels of etoposide and alopecia.

Cisplatin mainly reacts with N-7 of guanine and adenine to form adducts with the DNA (Kelland, 2007) resulting in the formation of intra and inter strands crosslinks, causing potential errors in DNA repair, resulting in accumulation of damaged DNA, and activation of apoptotic pathway in neoplastic and normal cells. Therefore, it was important to analyze both, the drug-metabolizing enzymes (GSTs) and the DNA damage repair proteins. We observed that *GSTT1*-null genotype is associated with lymphocytopenia (OR = 17.67, CI = 1.23–252.73, *p*-value = 0.034) and *GSTP1* rs1695 G/G genotype is associated with increased infections (OR = 12.25, CI = 1.05–143.09, *p*-value = 0.046). Similarly, *ERCC2* rs1799793 A/A genotype showed association with anemia (OR = 27.00, CI = 1.68–434.44, *p*-value = 0.020), *ERCC2* rs238406 A/A genotype was associated with leukopenia (OR = 5.5, CI = 1.26–24.10, *p*-value = 0.024) and *ERCC2* rs13181 T/G genotype was linked with alopecia (OR = 10.86, CI = 1.16–101.35, *p*-value = 0.036), indicating that defects in the metabolism and/or the response to cisplatin could lead to the specific severe ADRs (Table 3). This is in agreement with studies that report that G allele of *GSTP1* rs1695 has been associated with an increased risk of myelosuppression, polyneuropathy, and toxicity (Joerger et al., 2012). Conversely, it has been found that the genotype *GSTP1* A/A is predicted to show a suboptimal response to chemotherapy with fluorouracil/cisplatin, and a lower survival rate in patients with advanced gastric cancer (Ruzzo et al., 2006). Studies on *GSTM1* and *GSTT1* have shown that high expression levels of both enzymes result in a low response to chemotherapy, and deletion of these genes shows high degrees of toxicity (Bai et al., 1996; Ambrosone et al., 2001). In ovarian cancer patients, severe emesis grades III–IV were associated with *GSTT1*-null genotype (Khrunin et al., 2010). In contrast, in patients with *GSTM1*-null genotype the risk of thrombocytopenia and anemia was lower (Khrunin et al., 2010). For *GSTP1*, the G/G genotype seems to decrease the susceptibility to grade III neuropathy when compared to that in patients with A/G and/or A/A genotypes (recessive model of inheritance) in ovarian cancer (Khrunin et al., 2010). In this study, however, we did not find an association of these polymorphic enzymes with the above-mentioned adverse reactions. However, the effect of GST in hematological toxicity is reasonable, since GSTs are responsible for etoposide breakdown and elimination^2^.

The analysis of univariate associations among severe ADRs and non-genetic factors (Table 4) showed an association between cumulative dose of bleomycin, bleomycin dose by average and bleomycin cycles by range; and that these parameters are associated with febrile neutropenia, as well as cisplatin dose by range. Similarly, cumulative dose of etoposide (total or by average) is associated with leukopenia as well as the cumulative dose of cisplatin. Besides, neutropenia is associated with both, cumulative or cumulative by average dose of cisplatin or etoposide. These results are consistent with the studies that report the relationship between hematological ADRs and BEP drugs, particularly etoposide and cisplatin^5^^6^. Moreover, alopecia was significantly associated only with dose of bleomycin (cumulative and by average).

Multivariate analyses to obtain risk association models of severe ADRs, including polymorphisms and non-genetic variables (Table 5) yielded good models to partly explain febrile neutropenia (Pseudo R^2^: 0.0956), leukopenia (Pseudo R^2^: 0.1372), vomiting (Pseudo R^2^: 0.1231), nausea (Pseudo R^2^: 0.1278), and alopecia (Pseudo R^2^: 0.2135). Interestingly, in these models, only dosage but not the demographic variables were relevant for severe ADRs.

**FIGURE 1:**
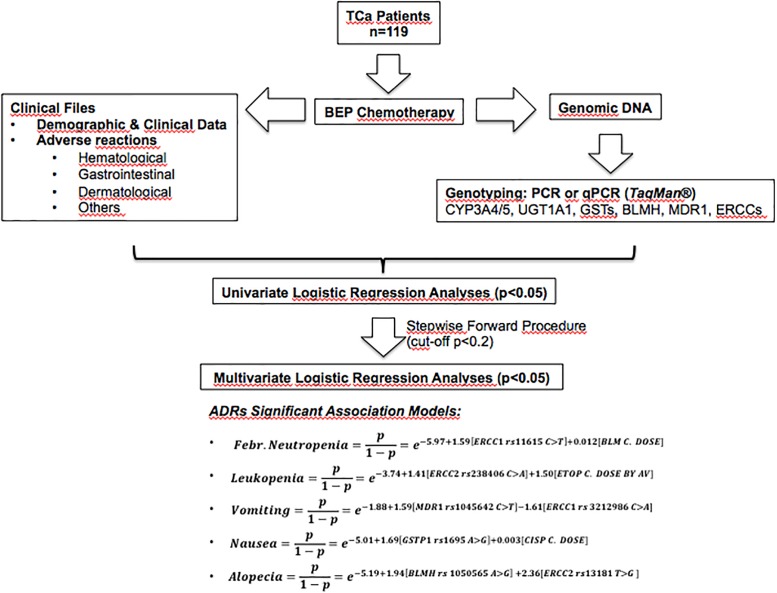
Scheme of this research and relevant results. BEP, bleomycin-etoposide-cisplatin therapy; ADRs, adverse drug reactions; TCa, testicular cancer.

For better understanding Figure 1 shows a scheme of this research and relevant results. After recruitment, genotyping and data collection from patients, genetic and non-genetic factors were submitted to logistic univariate statistical analyses. Then, logistic multivariate models were adjusted using a stepwise forward procedure with a cut-off *p*-value ≤ 0.2. The multivariate models are described by pseudo R^2^ values and equations for the obtained significant ADRs association models (febrile neutropenia, leukopenia, vomiting, nausea, and alopecia).

Despite our analysis, the study has some shortcomings. Although we had a relatively appropriate sample size for combinatorial analyses, fewer number of patients examined could mask potential associations, especially for low frequency polymorphisms, particularly in the multivariate analyses. Some other potentially candidate genes/polymorphisms were not evaluated in this study (based in level of evidence), which could be still relevant. Besides, the cumulative doses were obtained at the end of the therapy, which could limit our conclusions about this factor in relation to ADRs. We were not able to analyze additional potential toxicities such as hepatotoxicity and nephrotoxicity due to incomplete clinical data. These, and others missing clinical values, could be relevant, giving rise to a possible differential misclassification bias affecting estimated associations between potentially relevant combinations of risk factors and adverse reactions. Finally, we did not adjust *p*-values for multiple tests (Bonferroni’s adjustment), which could generate direct implications in α and the *p*-value. However, it has been proposed that the adjustment is not always recommended, especially when high number of comparison are performed and multiple false negatives need to be avoided, which is the case (Goldman, 2008; Kim and Bang, 2016).

## Conclusion

Our findings from the univariate analyses suggest that patients with *ERCC2* rs13181 T/G and/or *CYP3A4* rs2740574 A/G genotypes are more likely to develop grades III–IV alopecia; patients with *ERCC2* rs238406 C/C genotype may develop severe leukopenia; and patients with *GSTT1*-null genotype could develop lymphocytopenia. Patients with *ERCC2* rs1799793 A/A genotype were at higher risk of developing anemia. Patients with *BLMH* rs1050565 G/G genotype experienced severe pain, and patients with *GSTP1* G/G genotype were susceptible to severe infections. As expected, severe ADRs associated with non-genetic variables were hematological (neutropenia and leukopenia). The multivariate analyses showed an association between specific *ERCC1/2* genotypes and cumulative dose of BEP drugs with the appearance of severe leukopenia and/or febrile neutropenia. Grades III–IV vomiting, nausea and alopecia could also be partly explained by the presence of specific *ERCC1*/2, *MDR1*, *GSTP1*, and *BLMH* genotypes. Our study provides additional evidence for the use of pharmacogenetics as a useful tool for potential prediction of severe ADRs in testicular patients treated with BEP chemotherapy.

## Author Contributions

ML, JC, and NV: experimental analyses, analysis of data, and writing the manuscript. AR: experimental analyses and writing the manuscript. CS, LC, JR, RC, SM-M, and CC: experimental analyses. BC and KP: enrolment of patients. EG-M and JA: conception of the research. CA: analysis of data and enrolment of patients. DC: analysis of data. LQ: conception of the research, analysis of data, and writing the manuscript.

## Conflict of Interest Statement

The authors declare that the research was conducted in the absence of any commercial or financial relationships that could be construed as a potential conflict of interest.

## Footnotes

1 https://www.fda.gov/drugs/scienceresearch/ucm572698.htm

2 https://www.pharmgkb.org/pathway/PA2025

3 https://www.ncbi.nlm.nih.gov/snp

4 https://snpinfo.niehs.nih.gov

5 http://chemocare.com/chemotherapy/drug-info/etoposide.aspx

6 http://chemocare.com/chemotherapy/drug-info/cisplatin.aspx
