# Supplementary material for: Association Study Among Candidate Genetic Polymorphisms and Chemotherapy-Related Severe Toxicity in Testicular Cancer Patients
Source: Front Pharmacol. 2019 Mar 8;10:206. doi: 10.3389/fphar.2019.00206 (PMC6421934; doi:10.3389/fphar.2019.00206)
Supplement: Supplementary file 1 [file Table_1.docx]

**Supplementary Tables**

| **Table S1.** Candidate gene selection | | | |
| --- | --- | --- | --- |
|  | Gene | Polymorphism | References* |
| Cisplatin |  |  |  |
|  | GSTP1 | ^+^rs1695 | (Rednam, Scheurer, and Adesina 2012; Drögemöller et al. 2017) |
|  | GSTM1 | ^+^deletion | (Kap et al. 2014; Abbas et al. 2015; Pereira et al. 2016) |
|  | ERCC1 | ^+^rs11615 | (Ivanova 2012; Zucali et al. 2011; Tzvetkov et al. 2011; A V Khrunin et al. 2010) |
|  | ERCC1 | ^+^rs3212986 | (Tzvetkov et al. 2011; Andrey Khrunin et al. 2012; A V Khrunin et al. 2010) |
|  | ERCC2 | rs13181 | (Lehmann 2001; Clarkson and Wood 2005) |
|  | ERCC2 | rs1799793 | (Lehmann 2001; Clarkson and Wood 2005) |
|  | ERCC2 | rs238406 | (Lehmann 2001; Clarkson and Wood 2005) |
|  | GSTT1 | GSTT1- null | (Cho et al. 2010; Barahmani et al. 2009) |
| Bleomycin |  |  |  |
|  | BLMH | rs1050565 | (Bokemeyer 2008; De Haas et al. 2008) |
| Etoposide |  |  |  |
|  | MDR1 | rs1045642 | (Cizmarikova et al. 2010) |
|  | CYP3A4 | rs2740574 (*1B) | (Zhou et al. 2004; Felix et al. 1998) |
|  | CYP3A4 | rs35599367 (*22) | (Zhou et al. 2004; Felix et al. 1998) |
|  | CYP3A5 | rs776746 (*3) | (Zhou et al. 2004; Felix et al. 1998) |
|  | GSTM1 | GSTM1-null | (Cho et al. 2010; Barahmani et al. 2009; A V Khrunin et al. 2010) |
|  | GSTP1 | rs1695 | (Kishi et al. 2004) |
|  | GSTT1 | GSTT1- null | (Cho et al. 2010; Barahmani et al. 2009) |
|  | UGT1A1 | rs8175347 (*28) | (Anai et al. 2018; Ribrag et al. 2009) |
|  | ERCC1 | rs11615 | (Chiu et al. 2011; Tsai et al. 2011) |
|  | ERCC1 | rs3212986 | (Chiu et al. 2011; Tsai et al. 2011) |
|  | ERCC2 | rs13181 | (Lehmann 2001; Clarkson and Wood 2005) |
|  | ERCC2 | rs1799793 | (Lehmann 2001; Clarkson and Wood 2005) |
|  | ERCC2 | rs238406 | (Lehmann 2001; Clarkson and Wood 2005) |
| ^+^Level of evidence 1 or 2 in Pharmgkb database <https://www.pharmgkb.org> | | | |

*REFERENCES CHEMOTHERAPY

Abbas, Mohammad, Vandana Singh Kushwaha, Kirti Srivastava, and Monisha Banerjee. 2015. “Glutathione S-Transferase Gene Polymorphisms and Treatment Outcome in Cervical Cancer Patients under Concomitant Chemoradiation.” Edited by Ken Mills. *PLOS ONE* 10 (11): e0142501. doi:10.1371/journal.pone.0142501.

Anai, Satoshi, Eiji Iwama, Yasuto Yoneshima, Kohei Otsubo, Kentaro Tanaka, Yoichi Nakanishi, and Isamu Okamoto. 2018. “Association of Nephrotoxicity during Platinum-Etoposide Doublet Therapy with UGT1A1 Polymorphisms in Small Cell Lung Cancer Patients.” *Lung Cancer* 126 (December): 156–61. doi:10.1016/j.lungcan.2018.11.002.

Barahmani, Nadia, Sarah Carpentieri, Xio-Nan Li, Tao Wang, Yumei Cao, Laura Howe, Lindsay Kilburn, Murali Chintagumpala, Ching Lau, and M Fatih Okcu. 2009. “Glutathione S-Transferase M1 and T1 Polymorphisms May Predict Adverse Effects after Therapy in Children with Medulloblastoma.” *Neuro-Oncology* 11 (3): 292–300. doi:10.1215/15228517-2008-089.

Bokemeyer, Carsten. 2008. “Bleomycin in Testicular Cancer: Will Pharmacogenomics Improve Treatment Regimens?” *Journal of Clinical Oncology* 26 (11): 1783–85. doi:10.1200/JCO.2007.15.2991.

Chiu, Tai-Jan, Chang-Han Chen, Chih-Yen Chien, Shau-Hsuan Li, Hsin-Ting Tsai, and Yi-Ju Chen. 2011. “High ERCC1 Expression Predicts Cisplatin-Based Chemotherapy Resistance and Poor Outcome in Unresectable Squamous Cell Carcinoma of Head and Neck in a Betel-Chewing Area.” *Journal of Translational Medicine* 9 (1): 31. doi:10.1186/1479-5876-9-31.

Cho, Hyun-Jung, Hyeon-Seok Eom, Hyun-Ju Kim, In-Suk Kim, Gyeong Won Lee, and Sun-Young Kong. 2010. “Glutathione-S-Transferase Genotypes Influence the Risk of Chemotherapy-Related Toxicities and Prognosis in Korean Patients with Diffuse Large B-Cell Lymphoma.” *Cancer Genetics and Cytogenetics* 198 (1): 40–46. doi:10.1016/j.cancergencyto.2009.12.004.

Cizmarikova, M, M Wagnerova, L Schonova, V Habalova, a Kohut, a Linkova, M Sarissky, J Mojzis, L Mirossay, and a Mirossay. 2010. “MDR1 (C3435T) Polymorphism: Relation to the Risk of Breast Cancer and Therapeutic Outcome.” *The Pharmacogenomics Journal* 10 (1): 62–69. doi:10.1038/tpj.2009.41.

Clarkson, Stuart G, and Richard D Wood. 2005. “Polymorphisms in the Human XPD (ERCC2) Gene, DNA Repair Capacity and Cancer Susceptibility: An Appraisal.” *DNA Repair* 4 (10): 1068–74. doi:10.1016/j.dnarep.2005.07.001.

De Haas, Esther C., Nynke Zwart, Coby Meijer, Janine Nuver, H. Marike Boezen, Albert J.H. Suurmeijer, Harald J. Hoekstra, Gerrit Van Der Steege, Dirk Th. Sleijfer, and Jourik A. Gietema. 2008. “Variation in Bleomycin Hydrolase Gene Is Associated with Reduced Survival after Chemotherapy for Testicular Germ Cell Cancer.” *Journal of Clinical Oncology* 26 (11): 1817–23. doi:10.1200/JCO.2007.14.1606.

Drögemöller, Britt I., Jose G. Monzon, Amit P. Bhavsar, Adrienne E. Borrie, Beth Brooks, Galen E. B. Wright, Geoffrey Liu, et al. 2017. “Association Between *SLC16A5* Genetic Variation and Cisplatin-Induced Ototoxic Effects in Adult Patients With Testicular Cancer.” *JAMA Oncology* 3 (11): 1558. doi:10.1001/jamaoncol.2017.0502.

Felix, C A, A H Walker, B J Lange, T M Williams, N J Winick, N K Cheung, B D Lovett, P C Nowell, I A Blair, and T R Rebbeck. 1998. “Association of CYP3A4 Genotype with Treatment-Related Leukemia.” *Proceedings of the National Academy of Sciences of the United States of America* 95 (22): 13176–81. http://www.ncbi.nlm.nih.gov/pubmed/9789061.

Ivanova, Feodosia. 2012. “Pharmacogenomics of Cisplatin-Based Chemotherapy in Ovarian Cancer Patients of Different Ethnic Origins R Esearch A Rticle” 13: 171–78.

Kap, Elisabeth J, Swantje Richter, Anja Rudolph, Lina Jansen, Alexis Ulrich, Michael Hoffmeister, Cornelia M Ulrich, Hermann Brenner, and Jenny Chang-Claude. 2014. “Genetic Variants in the Glutathione S-Transferase Genes and Survival in Colorectal Cancer Patients after Chemotherapy and Differences According to Treatment with Oxaliplatin.” *Pharmacogenetics and Genomics* 24 (7). United States: 340–47. doi:10.1097/FPC.0000000000000059.

Khrunin, Andrey, Feodosia Ivanova, Alexey Moisseev, Denis Khokhrin, Yuliya Sleptsova, Vera Gorbunova, and Svetlana Limborska. 2012. “Pharmacogenomics of Cisplatin-Based Chemotherapy in Ovarian Cancer Patients of Different Ethnic Origins.” *Pharmacogenomics* 13 (2): 171–78. doi:10.2217/pgs.11.140.

Khrunin, A V, A Moisseev, V Gorbunova, and S Limborska. 2010. “Genetic Polymorphisms and the Efficacy and Toxicity of Cisplatin-Based Chemotherapy in Ovarian Cancer Patients.” *The Pharmacogenomics Journal* 10 (1). Nature Publishing Group: 54–61. doi:10.1038/tpj.2009.45.

Kishi, S., Wenjian Yang, Benoit Boureau, Stanislas Morand, Soma Das, Peixian Chen, Edwin H Cook, et al. 2004. “Effects of Prednisone and Genetic Polymorphisms on Etoposide Disposition in Children with Acute Lymphoblastic Leukemia.” *Blood* 103 (1): 67–72. doi:10.1182/blood-2003-06-2105.

Lehmann, a. R. 2001. “The Xeroderma Pigmentosum Group D (XPD) Gene: One Gene, Two Functions, Three Diseases.” *Genes & Development* 15 (1): 15–23. doi:10.1101/gad.859501.

Pereira, Deolinda, Joana Assis, Mónica Gomes, Augusto Nogueira, and Rui Medeiros. 2016. “Improvement of a Predictive Model in Ovarian Cancer Patients Submitted to Platinum-Based Chemotherapy: Implications of a GST Activity Profile.” *European Journal of Clinical Pharmacology* 72 (5): 545–53. doi:10.1007/s00228-016-2015-3.

Rednam, Surya, Michael E Scheurer, and Adekunle Adesina. 2012. “Glutathione S-Transferase P1 Single Nucleotide Polymorphism Predicts Permanent Ototoxicity in Children With Medulloblastoma,” no. September. doi:10.1002/pbc.

Ribrag, V., S. Koscielny, O. Casasnovas, C. Cazeneuve, P. Brice, F. Morschhauser, J. Gabarre, et al. 2009. “Pharmacogenetic Study in Hodgkin Lymphomas Reveals the Impact of UGT1A1 Polymorphisms on Patient Prognosis.” *Blood* 113 (14): 3307–13. doi:10.1182/blood-2008-03-148874.

Tsai, Min-Shao, Shao-Hsing Weng, Ya-Hsun Kuo, Yu-Fan Chiu, and Yun-Wei Lin. 2011. “Synergistic Effect of Curcumin and Cisplatin via Down-Regulation of Thymidine Phosphorylase and Excision Repair Cross-Complementary 1 (ERCC1).” *Molecular Pharmacology* 80 (1): 136–46. doi:10.1124/mol.111.071316.

Tzvetkov, Mladen V, Gerrit Behrens, Valerie P O’Brien, Karin Hohloch, Jürgen Brockmöller, and Peter Benöhr. 2011. “Pharmacogenetic Analyses of Cisplatin-Induced Nephrotoxicity Indicate a Renoprotective Effect of ERCC1 Polymorphisms.” *Pharmacogenomics* 12 (10): 1417–27. doi:10.2217/pgs.11.93.

Zhou, Wei, Sarada Gurubhagavatula, Geoffrey Liu, Sohee Park, Donna S Neuberg, John C Wain, Thomas J Lynch, and Li Su. 2004. “Excision Repair Cross-Complementation Group 1 Polymorphism Predicts Overall Survival in Advanced Non-Small Cell Lung Cancer Patients Treated With Platinum-Based Chemotherapy Excision Repair Cross-Complementation Group 1 Polymorphism Predicts Overall Survi.”

Zucali, P. A., E. Giovannetti, A. Destro, M. Mencoboni, G. L. Ceresoli, L. Gianoncelli, E. Lorenzi, et al. 2011. “Thymidylate Synthase and Excision Repair Cross-Complementing Group-1 as Predictors of Responsiveness in Mesothelioma Patients Treated with Pemetrexed/Carboplatin.” *Clinical Cancer Research* 17 (8): 2581–90. doi:10.1158/1078-0432.CCR-10-2873.

**Table S2.** a) Context sequences for *TaqMan®* probes and b) Primers for PCR genotyping used in this study

**a)**

| SNP | 5’🡪3’ Sequence* | N° Catalogue |
| --- | --- | --- |
| CYP3A4*1B (rs 2740574) | 5’-TAAAATCTATTAAATCGCCTCTCTC**[C/T]**TGCCCTTGTCTCTATGGCTGTCCTC-3’ | C_1837671_50 |
| CYP3A4*22 (rs 35599367) | 5’-GTGCCAGTGATGCAGCTGGCCCTAC**[G/A]**CTGGGTGTGATGGAGACACTGAACT-3’ | C_59013445_10 |
| CYP3A5*3  (rs 776746) | 5’-ATGTGGTCCAAACAGGGAAGAGATA**[T/C]**TGAAAGACAAAAGAGCTCTTTAAAG-3’ | C_26201809_30 |
| UGT1A1*28^Note^ | 5’-TTCTAAAAAGCCTTCTGTTTAATTT**[C/T]**TGGAAAAGAAGCCTAACTTGTTCAC-3’ | C_2669357_10 |
| BLMH  (rs 1050565) | 5’-CCCATGGGGTCCCATGCTGGCAGGA**[C/T]**AATGGGTTCCTGCTCTAACACAGCT-3’ | C_7473213_1_ |
| GSTP1  (rs 1695) | 5’-CGTGGAGGACCTCCGCTGCAAATAC**[A/G]**TCTCCCTCATCTACACCAACTATG-3’ | [C_3237198_20](https://www.thermofisher.com/order/genome-database/details/genotyping/C___3237198_20?CID=&ICID=&subtype=) |
| ERCC1  (rs 11615) | 5’-TTACGTCGCCAAATTCCCAGGGCAC**[A/G]**TTGCGCACGAACTTCAGTACGGGAT-3’ | C_2532959_1_ |
| ERCC1  (rs 3212986) | 5’-CACAGGCCGGGACAAGAAGCGGAAG**[A/C]**AGCAGCAGCAGCAGCCTGTGTAGTC-3’ | C_2532948_10 |
| ERCC2  (rs 13181) | 5’-TGCTGAGCAATCTGCTCTATCCTCT**[G/T]**CAGCGTCTCCTCTGATTCTAGCTGC-3’ | C_3145033_10 |
| ERCC2  (rs 238406) | 5’-CCTGCCCTCCAGTAACCTCATAGAA**[G/T]**CGGCAGTGGGGCAGGCTGGTGTCAT-3’ | C_8714009_10 |
| ERCC2  (rs 1799793) | 5’-CGGGGCTCACCCTGCAGCACTTCGT**[C/T]**GGGCAGCACGGGGTTGGCCAGGTGG-3’ | C_3145050_10 |
| MDR1  (rs 1045642) | 5’-TGTTGGCCTCCTTTGCTGCCCTCAC**[A/G]**ATCTCTTCCTGTGACACCACCCGGC-3’ | C_7586657_20 |

**The bracketed region indicates the position of the polymorphism in the gene sequence of the variants*

***Note:*** *UGT1A1* 28 (rs 8175347) was determined indirectly by the variant UGT1A1*80 (rs 887829) by TaqMan® probes****.***

**b)**

| Polymorphism | 5’🡪3’ Sequence | Amplicon (bp) |
| --- | --- | --- |
| GSTT1 reverse | 5´TCACCGGATCATGGCCAGCA3´ | 459 |
| GSTT1 forward | 5´TTCCTTACTGGTCCTCACATCTC3´ |  |
| β-Globin reverse | 5´GAAGAGCCAAGGACAGTTAC3´ | 268 |
| β- Globin forward | 5´CAACTTCATCCACGTTCACC3´ |  |
| GSTM1 reverse | 5´CTGGATTGTAGCAGATCATGC3´ | 273 |
| GSTM1 forward | 5´CTGCCCCACTTGATTGATGGG3´ |  |
| C44 reverse* | 5´CAGTGAAGAGGTGTAGCCGCT3 | 340 |
| C47 forward* | 5´TAGGAGTCTTGTCTCATGCCT 3´ |  |

**C44 and C47 primers for CYP1A1 amplification were used as amplification control band to determine GSTM1 deletion (Quiñones et al, 2001)*

**Table S3:** Polymorphisms characterization

|  | ***CYP3A4*1B*** | ***CYP3A5*3*** | ***UGT1A1*28*** | ***GSTP1*** | ***GSTM1*** | ***GSTT1*** | ***MDR1*** | ***ERCC1*** | ***ERCC2*** | ***BLMH*** |
| --- | --- | --- | --- | --- | --- | --- | --- | --- | --- | --- |
| **rs** | 2740574 | 776746 | 8175347 | 1695 | - | - | 1045642 | a) 11615  b) 3212986 | a) 238406  b) 13181  c) 1799793 | 1050565 |
| **Localization** | 5`promotor región | Intron 3 | TATA promotor | Exon 5 | Full Gen | Full Gen | Exon 26 | a) Exon 4  b) 3’-UTR region | a) Exon 6  b) Exon 23  c) Exon 10 | Exón 11 |
| **DNA alteration** | c.-392A> G | 6986A>G | TA (TA)7 | 313A>G | Null | Null | 3435C>T | a) 19007C>T  b) 8092C>A | a) 22541C>A  b) 35931T>G  c) 934G>A | 1450A>G |
| **Effect** | Transcriptional alteration (reduced) | Splicing defect | Transcriptional alteration (reduced) | p.Ile105Val | Deletion | Deletion | Alteration of conformational folding | a)Transcriptional alteration (reduced)  b) low mRNA stability | a) p.Arg156Arg Transcriptional alteration  b) p.Lys751Gln  c) p.Asp312Asn | p.Ile443Val |
| **Protein Effect** | Reduced activity | Reduced activity | Reduced activity | Reduced activity | No activity | No activity | Reduced activity | Reduced activity (a and b) | Reduced activity (a,b and c) | Reduced activity |

| T**able S4.** Genotype and allele frequencies in patients | | | | | | | | | | | |
| --- | --- | --- | --- | --- | --- | --- | --- | --- | --- | --- | --- |
|  | | | | | |  | | | | | |
| ***CYP3A4*1B (rs2740574)*** | **Genotype (n=119)** | | | **Allele** | | ***ERCC2 rs13181*** | **Genotype (n=115)** | | | **Allele** | |
| **Frequency** | A/A | A/G | G/G | F A | f G | **Frequency** | **T/T** | **T/G** | **G/G** | **f T** | **f G** |
| **No. (%)** | 106 (89.1) | 12 (10.1) | 1  (0.8) | 0.94 | 0.06 | **No. (%)** | 77  (67.0) | 32 (27.8) | 6  (5.2) | 0.81 | 0.19 |
|  | | | | | |  |  |  |  |  |  |
| ***CYP3A4*22 (rs35599367)*** | **Genotype (n=116)** | | | **Allele** | | ***ERCC2 rs1799793*** | **Genotype (n=114)** | | | **Allele** | |
| **Frequency** | C/C | C/T | T/T | f C | f T | **Frequency** | **G/G** | **A/G** | **A/A** | **f G** | **f A** |
| **No. (%)** | 113 (97.4) | 3  (2.6) | 0  (0) | 0.99 | 0.01 | **No. (%)** | 80 (70.2) | 30 (26.3) | 4  (3.5) | 0.83 | 0.17 |
|  | | | | | |  |  |  |  |  |  |
| ***CYP3A5*3 (rs776746)*** | **Genotype (n=116)** | | | **Allele** | | ***ERCC2 rs238406*** | **Genotype (114)** | | | **Allele** | |
| **Frequency** | A/A | A/G | G/G | f A | f G | **Frequency** | C/C | C/A | A/A | f C | f A |
| **No. (%)** | 1  (0.9) | 0  (0) | 115  (99.1) | 0.01 | 0.99 | **No. (%)** | 58 (50.9) | 30 (26.3) | 26 (22.8) | 0.64 | 0.36 |
|  | | | | | |  |  |  |  |  |  |
| ***UGT1A1*28 (rs8175347)**** | **Genotype (n=114)** | | | **Allele** | | ***MDR1 rs1045642*** | **Genotype (n=112)** | | | **Allele** | |
| **Frequency** | 6/6 | 6/7 | 7/7 | f 6 | f 7 | **Frequency** | C/C | C/T | T/T | f C | f T |
| **No. (%)** | 52  (45.6) | 53  (46.5) | 9  (7.9) | 0.69 | 0.31 | **No. (%)** | 31 (27.7) | 64 (57.1) | 17  (15.2) | 0.56 | 0.44 |
|  | | | | | |  |  |  |  |  |  |
| ***GSTM1(-)*** | **Genotype (n=109)** | | | **Allele** | | ***ERCC1 rs11615*** | **Genotype (n=108)** | | | **Allele** | |
| **Frequency** | No Null |  | Null | f wt | f (-) | **Frequency** | C/C | C/T | T/T | f C | f T |
| **No. (%)** | 68  (62.4) |  | 41  (37.6) | 0.62 | 0.38 | **No. (%)** | 23  (21.3) | 67 (62.0) | 18  (16.7) | 0.52 | 0.48 |
|  |  |  |  |  |  |  |  |  |  |  |  |
| ***GSTT1(-)*** | **Genotype (n=115)** | | | **Allele** | | ***ERCC1 rs3212986*** | **Genotype (n=117)** | | | **Allele** | |
| **Frequency** | No Null |  | Null | f wt | f (-) | **Frequency** | C/C | C/A | A/A | f C | f A |
| **No. (%)** | 110 (95.7) |  | 5  (4.3) | 0.96 | 0.04 | **No. (%)** | 52 (44.4) | 56 (47.9) | 9  (7.7) | 0.68 | 0.32 |
|  |  |  |  |  |  |  |  |  |  |  |  |
| ***GSTP1 rs1695*** | **Genotype (n=117)** | | | **Allele** | | ***BLMH rs1050565*** | **Genotype (n=119)** | | | **Allele** | |
| **Frequency** | A/A | A/G | G/G | f A | f G | **Frequency** | A/A | A/G | G/G | f A | f G |
| **No. (%)** | 39  (33.3) | 60  (51.3) | 18  (15.4) | 0.59 | 0.41 | **No. (%)** | 29 (24.4) | 64 (53.8) | 26  (21.8) | 0.51 | 0.49 |

***Note****: UGT1A1*80 (rs887829) was used to indirectly determine UGT1A1*28 (rs 8175347), because they are in linkage disequilibrium. (*[*https://www.ncbi.nlm.nih.gov/pmc/articles/PMC4785051/*](https://www.ncbi.nlm.nih.gov/pmc/articles/PMC4785051/)*)*

*Some patients in some polymorphisms were not genotyped due to DNA shortage.*

| **Table S5.** Univariate logistic regression analysis of risk of severe ADRs (III-IV)  according to genotypes | | | | | |  |
| --- | --- | --- | --- | --- | --- | --- |
| ADR* | n | OR** | 95% IC*** | | p-value**** |  |
| **Hematological ADRs** | | | | | |  |
| FEBRILE NEUTROPENIA | | |  | |  |  |
| ERCC2 (rs 13181) |  |  |  | |  |  |
| T/T | 77 | 1.00 |  | | Reference |  |
| T/G | 32 | 2.31 | 0.71-7.51 | | 0.165 |  |
| G/G | 6 | 5.00 | 0.77-32.34 | | 0.091 |  |
| ERCC2 (rs 13181) |  |  |  | |  |  |
| T/T + T/G | 109 | 1.00 |  | | Reference |  |
| G/G | 6 | 3.69 | 0.61-22.19 | | 0.153 |  |
| ERCC2 (rs 13181) |  |  |  | |  |  |
| T/T | 77 | 1.00 |  | | Reference |  |
| T/G + G/G | 38 | 2.67 | 0.89-8.02 | | 0.081 |  |
| ERCC1 (rs 11615) |  |  |  | |  |  |
| C/C | 23 | 1.00 |  | | Reference |  |
| C/T | 67 | 0.38 | 0.09-1.57 | | 0.183 |  |
| T/T | 18 | 1.36 | 0.29-6.38 | | 0.699 |  |
| ERCC1 (rs 11615) |  |  | |  | |  |
| C/C + C/T | 90 | 1.00 |  | | Reference |  |
| T/T | 18 | 2.58 | 0.70-9.50 | | 0.157 |  |
| ERCC1 (rs 3212986) |  |  |  | |  |  |
| C/C | 52 | 1.00 |  | | Reference |  |
| C/A | 56 | 0.92 | 0.28-3.06 | | 0.892 |  |
| A/A | 9 | 3.83 | 0.75-19.49 | | 0.105 |  |
| ERCC1 (rs 3212986) |  |  |  | |  |  |
| C/C + C/A | 108 | 1.00 |  | | Reference |  |
| A/A | 9 | 4.00 | 0.88-18.11 | | 0.072 |  |
| ANEMIA |  |  |  | |  |  |
| ERCC2 (rs 13181) |  |  |  | |  |  |
| T/T + T/G | 107 | 1.00 |  | | Reference |  |
| G/G | 6 | 10.50 | 0.81-136.18 | | 0.072 |  |
| ERCC2 (rs 1799793) |  |  |  | |  |  |
| G/G + G/A | 110 | 1.00 |  | | Reference |  |
| A/A | 3 | 27.00 | 1.68-434.44 | | 0.020 |  |
| ERCC2 (rs 1799793) |  |  |  | |  |  |
| G/G | 80 | 1.00 |  | | Reference |  |
| G/A + A/A | 33 | 5.10 | 0.45-58.25 | | 0.190 |  |
| LEUKOPENIA |  |  |  | |  | |
| GSTT1 |  |  |  | |  | |
| No Null | 109 | 1.00 |  | | Reference | |
| Null | 5 | 4.92 | 0.75-32.28 | | 0.097 | |
| ERCC2 (rs 13181) |  |  |  | |  | |
| T/T + T/G | 108 | 1.00 |  | | Reference | |
| G/G | 6 | 4.00 | 0.66-24.21 | | 0.131 | |
| ERCC2 (rs 238406) |  |  |  | |  | |
| C/C | 58 | 1.00 |  | | Reference | |
| C/A | 29 | 3.82 | 0.84-17.28 | | 0.082 | |
| A/A | 26 | 5.50 | 1.26-24.10 | | 0.024 | |
| ERCC2 (rs 238406) |  |  |  | |  | |
| C/C + C/A | 87 | 1.00 |  | | Reference | |
| A/A | 26 | 2.96 | 0.92-9.51 | | 0.068 | |
| ERCC2 (rs 238406) |  |  |  | |  | |
| C/C | 58 | 1.00 |  | | Reference | |
| C/A + A/A | 55 | 4.58 | 1.20-17.45 | | 0.026 | |
| MDR1 (rs 1045642) |  |  |  | |  | |
| CC | 31 | 1.00 |  | | Reference | |
| CT | 64 | 0.29 | 0.08-1.01 | | 0.051 | |
| TT | 17 | 0.46 | 0.08-2.50 | | 0.366 | |
| MDR1 (rs 1045642) |  |  |  | |  | |
| CC | 31 | 1.00 |  | | Reference | |
| CT + TT | 81 | 0.32 | 0.10-1.02 | | 0.054 | |
| NEUTROPENIA |  |  |  | |  | |
| UGT1A1*28 (rs 8175347) |  |  |  | |  | |
| 6/6 | 51 | 1.00 |  | | Reference | |
| 6/7 | 51 | 1.78 | 0.80-3.96 | | 0.159 | |
| 7/7 | 9 | 1.60 | 0.38-6.74 | | 0.522 | |
| UGT1A1*28 (rs 8175347) |  |  |  | |  | |
| 6/6 | 51 | 1.00 |  | | Reference | |
| 6/7 + 7/7 | 60 | 1.75 | 0.81-3.79 | | 0.155 | |
| ERCC2 (rs 13181) |  |  |  | |  | |
| T/T | 74 | 1.00 |  | | Reference | |
| T/G | 32 | 1.12 | 0.48-2.62 | | 0.787 | |
| G/G | 6 | 3.29 | 0.56-19.12 | | 0.186 | |
| ERCC2 (rs 13181) |  |  |  | |  | |
| T/T + T/G | 106 | 1.00 |  | | Reference | |
| G/G | 6 | 3.17 | 0.56-18.10 | | 0.194 | |
| ERCC2 (rs 1799793) |  |  |  | |  | |
| G/G | 78 | 1.00 |  | | Reference | |
| G/A | 30 | 0.76 | 0.31-1.84 | | 0.539 | |
| A/A | 4 | 4.55 | 0.45-45.74 | | 0.198 | |
| ERCC2 (rs 1799793) |  |  |  | |  | |
| G/G + G/A | 108 | 1.00 |  | | Reference | |
| A/A | 4 | 4.90 | 0.49-48.72 | | 0.175 | |
| LYMPHOCYTOPENIA |  |  |  | |  |  |
| UGT1A1*28 (rs 8175347) |  |  |  | |  |  |
| 6/6 + 6/7 | 102 | 1.00 |  | | Reference |  |
| 7/7 | 9 | 6.25 | 0.51-76.61 | | 0.152 |  |
| GSTT1 |  |  |  | |  |  |
| No Null | 108 | 1.00 |  | | Reference |  |
| Null | 4 | 17.67 | 1.23-252.73 | | 0.034 |  |
| ERCC2 (rs 238406) |  |  |  | |  |  |
| C/C + C/A | 85 | 1.00 |  | | Reference |  |
| A/A | 26 | 7.00 | 0.61-80.55 | | 0.118 |  |
| ERCC1 (rs 3212986) |  |  |  | |  |  |
| C/C + C/A | 105 | 1.00 |  | | Reference |  |
| A/A | 9 | 6.44 | 0.53-78.89 | | 0.145 |  |
| **Gastrointestinal ADRs** | | | | | |  |
| VOMITING |  |  |  | |  |  |
| MDR1 (rs 1045642) |  |  |  | |  |  |
| CC + CT | 95 | 1.00 |  | | Reference |  |
| TT | 17 | 3.87 | 0.99-15.06 | | 0.051 |  |
| ERCC2 (rs 238406) |  |  |  | |  |  |
| C/C | 58 | 1.00 |  | | Reference |  |
| C/A | 30 | 2.70 | 0.67-10.92 | | 0.164 |  |
| A/A | 26 | 1.13 | 0.19-6.57 | | 0.896 |  |
| ERCC1 (rs 3212986) |  |  |  | |  |  |
| C/C | 52 | 1.00 |  | | Reference |  |
| C/A | 56 | 0.20 | 0.04-1.01 | | 0.051 |  |
| A/A | 9 | 0.69 | 0.08-6.27 | | 0.740 |  |
| ERCC1 (rs 3212986) |  |  |  | |  |  |
| C/C | 52 | 1.00 |  | | Reference |  |
| C/A + A/A | 65 | 0.27 | 0.07-1.06 | | 0.060 |  |
| NAUSEA |  |  |  | |  |  |
| CYP3A4*1B (rs 2740574) |  |  |  | |  |  |
| A/A | 106 | 1.00 |  | | Reference |  |
| A/G | 12 | 3.33 | 0.59-18.75 | | 0.172 |  |
| G/G | 1 | - |  | |  |  |
| UGT1A1*28 (rs 8175347) |  |  |  | |  |  |
| 6/6 | 52 | 1.00 |  | | Reference |  |
| 6/7 | 53 | 3.19 | 0.61-16.60 | | 0.168 |  |
| 7/7 | 9 | - |  | |  |  |
| GSTP1 (rs 1695) |  |  |  | |  |  |
| A/A + A/G | 99 | 1.00 |  | | Reference |  |
| G/G | 18 | 3.76 | 0.81-17.39 | | 0.090 |  |
| ERCC2 (rs 13181) |  |  |  | |  |  |
| T/T | 77 | 1.00 |  | | Reference |  |
| T/G | 32 | 3.52 | 0.74-16.75 | | 0.113 |  |
| G/G | 6 | 4.93 | 0.43-56.47 | | 0.199 |  |
| ERCC2 (rs 13181) |  |  |  | |  |  |
| T/T | 77 | 1.00 |  | | Reference |  |
| T/G + G/G | 38 | 3.74 | 0.84-16.57 | | 0.083 |  |
| **Other ADRs** | | | | | |  |
| ALOPECIA |  |  |  | |  |  |
| CYP3A4*1B (rs 2740574) |  |  |  | |  |  |
| A/A | 106 | 1.00 |  | | Reference |  |
| A/G | 12 | 6.87 | 1.02-46.06 | | 0.047 |  |
| G/G | 1 | - |  | |  |  |
| CYP3A4*1B (rs 2740574) |  |  |  | |  |  |
| A/A | 106 | 1.00 |  | | Reference |  |
| A/G + G/G | 13 | 6.24 | 0.94-41.49 | | 0.058 |  |
| GSTP1 (rs 1695) |  |  |  | |  |  |
| A/A + A/G | 99 | 1.00 |  | | Reference |  |
| G/G | 18 | 6.06 | 0.80-46.16 | | 0.082 |  |
| BLMH (rs 1050565) |  |  |  | |  |  |
| A/A + A/G | 93 | 1.00 |  | | Reference |  |
| G/G | 26 | 5.93 | 0.94-37.62 | | 0.059 |  |
| ERCC2 (rs 13181) |  |  |  | |  |  |
| T/T | 77 | 1.00 |  | | Reference |  |
| T/G | 32 | 10.86 | 1.16-101.35 | | 0.036 |  |
| G/G | 6 | - |  | |  |  |
| ERCC2 (rs 13181) |  |  |  | |  |  |
| T/T | 77 | 1.00 |  | | Reference |  |
| T/G + G/G | 38 | 8.94 | 0.96-83.01 | | 0.054 |  |
| PAIN |  |  |  | |  |  |
| BLMH (rs 1050565) |  |  |  | |  |  |
| A/A + A/G | 93 | 1.00 |  | | Reference |  |
| G/G | 26 | 16.73 | 1.78-157.15 | | 0.014 |  |
| NEUROTOXICITY | | | | | |  |
| ERCC1 (rs 3212986) |  |  |  | |  |  |
| C/C + C/A | 108 | 1.00 |  | | Reference |  |
| A/A | 9 | 13.38 | 0.76-234.31 | | 0.076 |  |
| INFECTIONS |  |  |  | |  |  |
| GSTP1 (rs 1695) |  |  |  | |  |  |
| A/A + A/G | 99 | 1.00 |  | | Reference |  |
| G/G | 18 | 12.25 | 1.05-143.09 | | 0.046 |  |
| ERCC2 (rs 13181) |  |  |  | |  |  |
| T/T | 77 | 1.00 |  | | Reference |  |
| T/G | 32 | 5.07 | 0.44-57.98 | | 0.192 |  |
| G/G | 6 | - |  | |  |  |
| ERCC2 (rs 1799793) |  |  |  | |  |  |
| G/G | 80 | 1.00 |  | | Reference |  |
| G/A | 30 | 5.64 | 0.49-64.67 | | 0.164 |  |
| A/A | 4 | - |  | |  |  |
| ERCC1 (rs 3212986) |  |  |  | |  |  |
| C/C + C/A | 108 | 1.00 |  | | Reference |  |
| A/A | 9 | 6.625 | 0.54-81.17 | | 0.139 |  |

**ADR: adverse drug reaction, evaluated with CTCAEv5.0*

***OR: odds ratio*

****95% CI: 95% confidence interval*

***** Only p-values ≤ 0.20 are included, to be incorporated to multivariate analyses*

*Note: UGT1A1*80 (rs887829) was used to indirectly determine UGT1A1*28 (rs 8175347), because they are in linkage disequilibrium. (*[*https://www.ncbi.nlm.nih.gov/pmc/articles/PMC4785051/*](https://www.ncbi.nlm.nih.gov/pmc/articles/PMC4785051/)*)*

| **Table S6.** Univariate logistic regression analysis of risk of high grade ADRs (III-IV) according to non-genetic factors. | | | | |
| --- | --- | --- | --- | --- |
| ADR* | n | OR** | 95% IC*** | p-value**** |
| **Hematological ADRs** |  |  |  |  |
| FEBRILE NEUTROPENIA |  |  |  |  |
| WEIGHT | 115 | 0.96 | 0.91-1.01 | 0.086 |
| CHEMOTHERAPY CYCLES |  |  |  |  |
| ≤2 | 31 | 1.00 |  | Reference |
| >2 | 88 | 5.68 | 0.71-45.10 | 0.101 |
| CISPLATIN CUMULATIVE DOSE | 117 | 1.00 | 1.00-1.00 | 0.109 |
| BLEOMYCIN CUMULATIVE DOSE | 117 | 1.01 | 1.00-1.02 | 0.014 |
| CISPLATIN CUMULATIVE DOSE BY AVERAGE |  |  |  |  |
| ≤average | 59 | 1.00 |  | Reference |
| >average | 58 | 3.22 | 0.96-10.78 | 0.058 |
| ETOPOSIDE CUMULATIVE DOSE BY AVERAGE |  |  |  |  |
| ≤average | 63 | 1.00 |  | Reference |
| >average | 54 | 2.64 | 0.84-8.27 | 0.096 |
| BLEOMYCIN CUMULATIVE DOSE BY AVERAGE |  |  |  |  |
| ≤average | 81 | 1.00 |  | Reference |
| >average | 36 | 3.02 | 1.00-9.11 | 0.050 |
| CHEMOTHERAPY CYCLES |  |  |  |  |
| 1-2 | 31 | 1.00 |  | Reference |
| 3 | 49 | 3.41 | 0.38-30.66 | 0.274 |
| 4-5 | 39 | 9.00 | 1.07-75.51 | 0.043 |
| LEUKOPENIA |  |  |  |  |
| AGE | 118 | 1.06 | 1.00-1.13 | 0.054 |
| AGE BY AVERAGE |  |  |  |  |
| ≤average | 73 | 1.00 |  | Reference |
| >average | 45 | 2.79 | 0.92-8.47 | 0.070 |
| CHEMOTHERAPY CYCLES |  |  |  |  |
| ≤2 | 30 | 1.00 |  | Reference |
| >2 | 88 | 5.49 | 0.69-43.64 | 0.108 |
| CISPLATIN CUMULATIVE DOSE | 116 | 1.00 | 1.00-1.01 | 0.017 |
| ETOPOSIDE CUMULATIVE DOSE | 116 | 1.00 | 1.00-1.00 | 0.006 |
| BLEOMYCIN CUMULATIVE DOSE | 116 | 1.01 | 1.00-1.01 | 0.147 |
| CISPLATIN CUMULATIVE DOSE BY AVERAGE |  |  |  |  |
| ≤average | 58 | 1.00 |  | Reference |
| >average | 58 | 2.21 | 9.070-6.92 | 0.174 |
| ETOPOSIDE CUMULATIVE DOSE BY AVERAGE |  |  |  |  |
| ≤average | 62 | 1.00 |  | Reference |
| >average | 54 | 5.62 | 1.49-21.15 | 0.011 |
| CHEMOTHERAPY CYCLES |  |  |  |  |
| 1-2 | 30 | 1.00 |  | Reference |
| 3 | 49 | 4.83 | 0.56-41.41 | 0.151 |
| 4-5 | 39 | 6.34 | 0.74-54.72 | 0.093 |
| NEUTROPENIA |  |  |  |  |
| CHEMOTHERAPY CYCLES |  |  |  |  |
| ≤2 | 30 | 1.00 |  | Reference |
| >2 | 86 | 2.18 | 0.87-5.43 | 0.095 |
| CISPLATIN CUMULATIVE DOSE | 114 | 1.00 | 1.00-1.00 | 0.037 |
| ETOPOSIDE CUMULATIVE DOSE | 114 | 1.00 | 1.00-1.00 | 0.041 |
| BLEOMYCIN CUMULATIVE DOSE | 114 | 1.01 | 1.00-1.01 | 0.077 |
| CISPLATIN CUMULATIVE DOSE BY AVERAGE |  |  |  |  |
| ≤average | 58 | 1.00 |  | Reference |
| >average | 56 | 2.82 | 1.29-6.14 | 0.009 |
| ETOPOSIDE CUMULATIVE DOSE BY AVERAGE |  |  |  |  |
| ≤average | 60 | 1.00 |  | Reference |
| >average | 54 | 2.72 | 1.26-5.91 | 0.011 |
| CHEMOTHERAPY CYCLES |  |  |  |  |
| 1-2 | 30 | 1.00 |  | Reference |
| 3 | 47 | 1.87 | 0.69-5.06 | 0.220 |
| 4-5 | 39 | 2.61 | 0.94-7.28 | 0.066 |
| **Gastrointestinal ADRs** |  |  |  |  |
| VOMITING |  |  |  |  |
| AGE BY AVERAGE |  |  |  |  |
| ≤average | 73 | 1.00 |  | Reference |
| >average | 46 | 0.32 | 0.07-1.57 | 0.161 |
| HEIGHT BY AVERAGE |  |  |  |  |
| ≤average | 57 | 1.00 |  | Reference |
| >average | 57 | 2.52 | 0.62-10.28 | 0.198 |
| NAUSEA |  |  |  |  |
| AGE BY AVERAGE |  |  |  |  |
| ≤average | 73 | 1.00 |  | Reference |
| >average | 46 | 2.85 | 0.65-12.53 | 0.167 |
| CISPLATIN CUMULATIVE DOSE | 117 | 1.00 | 1.00-1.01 | 0.068 |
| **Other ADRs** |  |  |  |  |
| ALOPECIA |  |  |  |  |
| CUMULATIVE BLEOMYCIN DOSE | 117 | 1.02 | 1.00-1.03 | 0.046 |
| CISPLATIN CUMULATIVE DOSE BY AVERAGE |  |  |  |  |
| ≤average | 59 | 1.00 |  | Reference |
| >average | 58 | 4.30 | 0.47-39.65 | 0.199 |
| BLEOMYCIN CUMULATIVE DOSE BY AVERAGE |  |  |  |  |
| ≤average | 81 | 1.00 |  | Reference |
| >average | 36 | 10.00 | 1.08-92.94 | 0.043 |

**ADR: adverse drug reaction, evaluated with CTCAEv5.0*

***OR: odds ratio*

****95% CI: 95% confidence interval*

***** Only p-values ≤ 0.20 are included, to be incorporated to multivariate analyses*
